# Meta-analysis of the responses of tree and herb to elevated CO_2_ in Brazil

**DOI:** 10.1038/s41598-023-40783-5

**Published:** 2023-09-22

**Authors:** Janaina da Silva Fortirer, Adriana Grandis, Débora Pagliuso, Camila de Toledo Castanho, Marcos Silveira Buckeridge

**Affiliations:** 1 Laboratório de Fisiologia Ecológica de Plantas, Lafieco, Botany Department, Biosciences Institute at University of São Paulo, São Paulo, Brazil; 2 Departamento de Ciências Ambientais, Universidade Federal de São Paulo, São Paulo, Brazil

**Keywords:** Biochemistry, Plant sciences, Climate sciences, Environmental sciences

## Abstract

The CO_2_ concentration has increased in the atmosphere due to fossil fuel consumption, deforestation, and land-use changes. Brazil represents one of the primary sources of food on the planet and is also the world's largest tropical rainforest, one of the hot spots of biodiversity in the world. In this work, a meta-analysis was conducted to compare several CO_2_ Brazilian experiments displaying the diversity of plant responses according to life habits, such as trees (79% natives and 21% cultivated) and herbs (33% natives and 67% cultivated). We found that trees and herbs display different responses. The young trees tend to allocate carbon from increased photosynthetic rates and lower respiration in the dark—to organ development, increasing leaves, roots, and stem biomasses. In addition, more starch is accumulated in the young trees, denoting a fine control of carbon metabolism through carbohydrate storage. Herbs increased drastically in water use efficiency, controlled by stomatal conductance, with more soluble sugars, probably with a transient accumulation of carbon primarily stored in seeds as a response to elevated CO_2_.

## Introduction

The carbon dioxide (CO_2_) concentration has increased from ~ 280 to ~ 415 ppm in the atmosphere due to fossil fuel consumption, deforestation, and land-use changes^1–5^. The Intergovernmental Panel on Climate Change (IPCC) stated that by 2100, CO_2_ levels might reach the 1300 ppm mark^2^ and consequently increase the global temperature, needing mitigation alternatives to restrain climate change. The IPCC's 2021 report provides valuable insights into the potential impacts of elevated CO_2_ concentrations on plants, which can significantly improve plant growth and development. It highlights the need for further research to understand better the complex interactions between CO_2_ and other climate change factors and their effects on plant physiology, growth, and ecosystem functioning^5^.

One of the manners to capture the CO_2_ is forest maintenance and planting trees for carbon assimilation and biomass accumulation^6^. The increase in CO_2_ concentration stimulates photosynthesis, resulting in a productivity gain and more carbon storage^7–11^.

The photosynthesis parameters affected when plants grow under elevated CO_2_ (eCO_2_) are the reduction in stomatal conductance, leaf dark respiration rate, transpiration rate, maximum Rubisco enzyme carboxylation rate, and maximum transport of electrons rate that results in carbon assimilation increase^2,6,12–18^. The increase in CO_2_ concentration can stimulate photosynthesis in plants; consequently, the stimulation is influenced by various processes such as carboxylation and product synthesis^19^. The rate of photosynthesis can be controlled by Rubisco, which is sensitive to CO_2_, and other less sensitive components^19^. In maize leaves, eCO_2_ concentration decreased whole-leaf chlorophyll and protein content^20^. The stomatal index was also significantly increased in plants grown at high CO_2_ concentrations^20^. Furthermore, eCO_2_ reduced transpiration and water consumption in tomato plants, increasing water use efficiency^21^ and decreasing leaf transpiration rates^22^. The increase in leaf dark respiration can result from the direct instantaneous effect of increased CO_2_ concentration and the longer-term indirect effect due to changes in leaf composition^23^. The increased carbon assimilation resulting from elevated CO_2_ concentrations has enhanced different crop species' growth, productivity, and biochemical constituents^24^. In leguminous plants, eCO_2_ concentrations have increased chlorophyll, total starch, sucrose, and total carbohydrate content^25^.

Exposure to eCO_2_ can lead to various biochemical changes in plants, including photosynthesis, respiration, chlorophyll content, and starch accumulation^26^. Yelle et al.^27^ investigated the acclimation of tomato plants to eCO_2_, which observed an accumulation of starch in the chloroplasts. This suggests that starch alone can not fully explain the loss of photosynthetic efficiency in eCO_2_-grown plants. To understand the biological adaptations to abiotic stress, such as eCO_2_, it is important to select crops to verify their impact on plant development^26^.

Several publications have widely identified changes in these parameters so that large amounts of data can be compiled to provide panels for understanding the climate change effect on plants. One way to analyze and summarize the data is meta-analysis, which affords a comparative analysis of several eCO_2_ experiments displaying the diversity of plant responses according to life habits such as trees and herbs^15,28^. Performing meta-analyses with data on leaf photosynthesis of forest trees and crops is important because such data are essential for modeling the future of carbon storage and sequestration on the planet^29,30^ and also the future changes in agriculture and food production^31,32^.

The available data, including meta-analyses, is overwhelmed with temperate climate species^6,12,13,18,28,33–35^, lacking data from tropical (cultivated/exotic or native) plants^6,28,35–38^ and preventing more accurate analysis of some key regions containing high biodiversity and food production in the world. Contrasting to the high proportion of publications focusing on temperate species, about 43% of all Earth's tree species occur in South America^39^, with tropical and subtropical plants allocating 52% of the carbon on Earth's surface to biomass storage^6,35^.

A recent meta-analysis about productivity and its potential for crop adaptation under eCO_2_ included a single study from Brazil with coffee trees^35^. However, considering that Brazil represents one of the primary sources of food on the planet^40^ and is also the world's largest area of tropical rainforest^35^, representing one of the hot spots of biodiversity in the world, it would be essential to include studies performed in the region to obtain a general and accurate view of the effects of CO_2_ elevation for food production and biodiversity.

Plants in elevated CO_2_ environments in neotropical regions are of great interest due to their importance for understanding the response of these plants to changes in atmospheric conditions. Levy-Varon et al.^41^ investigated how symbiotic nitrogen fixation influences the tropical forest carbon sink. They found that planted trees can double carbon accumulation early in succession and increase total carbon in mature forests by approximately 10%. It is important to consider the diversity of functional plant communities in understanding the carbon sequestration potential of neotropical plants at eCO_2_. Rull and Vegas-Vilarrúbia^42^ performed simulations involving all known vascular flora of the neotropical Guayana Highlands and predicted the potential extinction of approximately 80% of species due to global warming by the end of this century. Despite these studies, there is still a gap in knowledge about the response of neotropical plants to elevated CO_2_. Studies about the effects of eCO_2_ on Brazilian plants have been carried out in the last couple of decades^16,43–46^, and it has been recently pointed out that such data remain a gap in meta-analysis works^42^.

This work aimed to perform a meta-analysis on the eCO_2_ responses in plant physiological parameters in Brazilian climates, representing a relevant portion of the neotropics. In these analyses, it was possible to: (I) estimate the size of the average effects of high atmospheric CO_2_ on biomass, biochemical, and photosynthesis parameters and (II) verify whether the eCO_2_ effects are influenced by the species' life habits (trees and herbs) with the hypothesis that trees and herbs would respond differently to elevated CO_2_ concentrations.

## Results

### Photosynthetic parameters, biomass, and starch increased in leaves of tropical plants under elevated CO_2_

**Figure 1:**
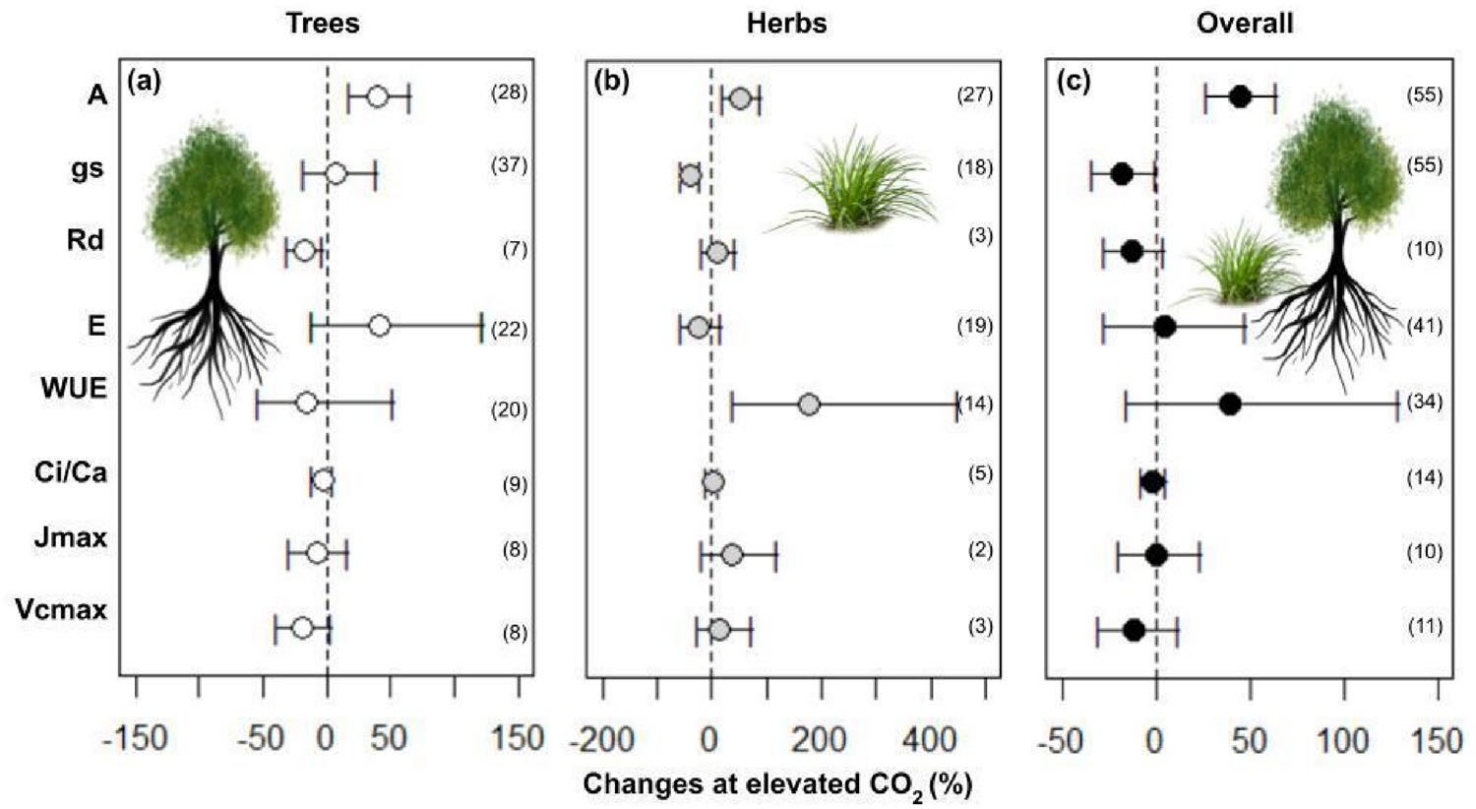
Responses of photosynthetic variables: Net CO_2_ assimilation (A), stomatal conductance (gs), dark respiration (Rd), foliar transpiration (E), water use efficiency (WUE), intercellular/ambient CO_2_ rate (Ci/Ca), maximum electron transport rate (J_max_), and maximum Rubisco carboxylation rate (Vc_max_) according to life habits: Trees (**a**), Herbs (**b**), and Overall (**c**) in plants grown in elevated CO_2_. The circles represent the percentage changes in elevated CO_2_. Error bars represent 95% confidence intervals. Study numbers for each variable are shown in parentheses.

**Figure 2:**
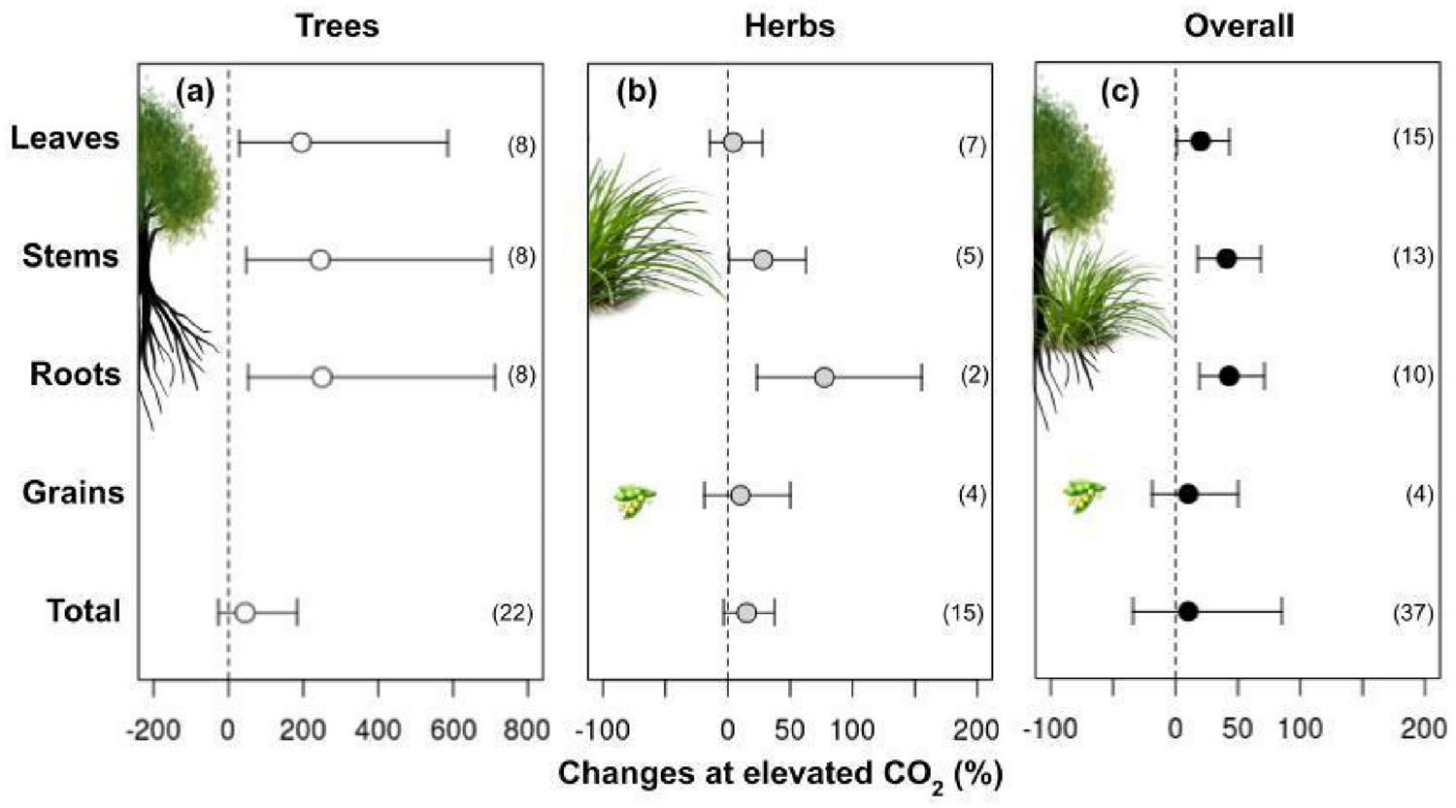
Biomass responses in each plant organ (leaf, stem, root, grain, and total) in plants grown into elevated CO_2_ according to life habits: Trees (**a**), Herbs (**b**), and Overall (**c**). The circles represent the percentage changes in elevated CO_2_. Error bars represent 95% confidence intervals. Study numbers for each variable are shown in parentheses.

**Figure 3:**
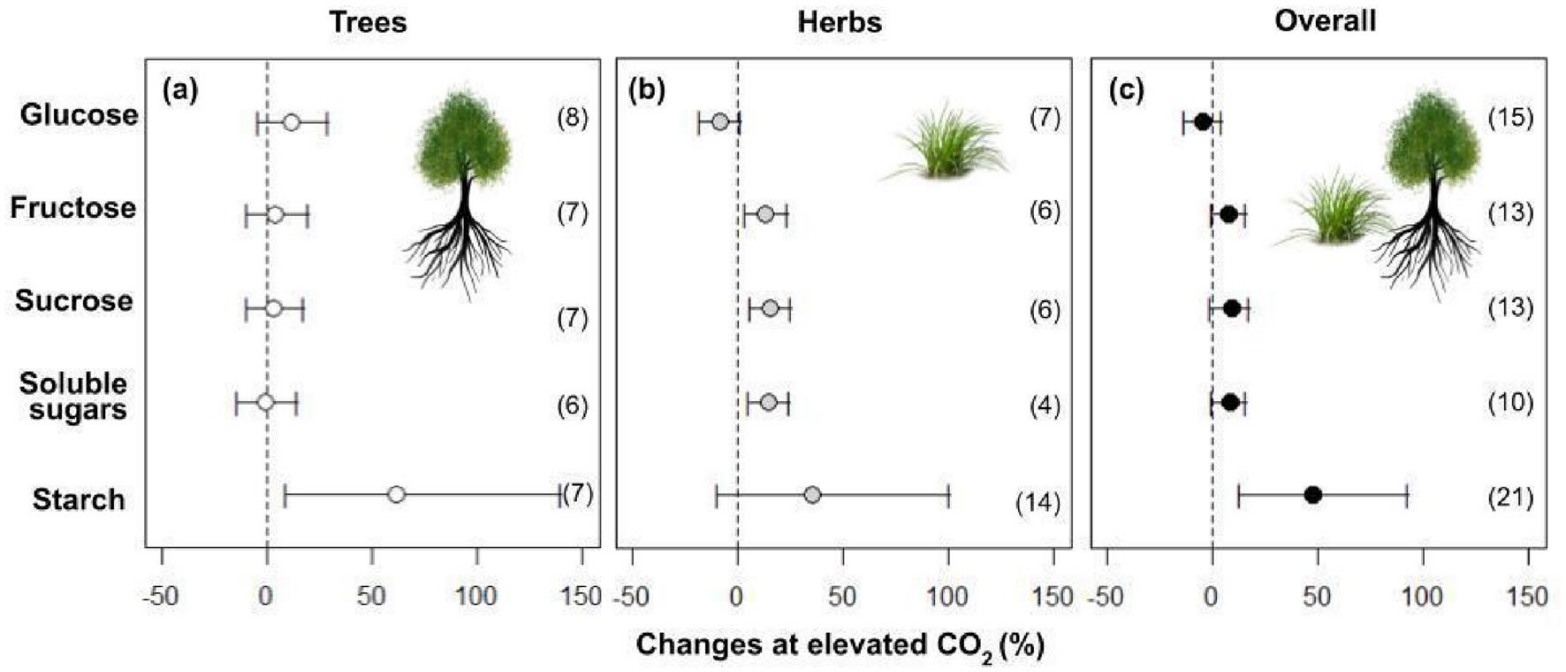
Responses of non-structural carbohydrates (glucose, fructose, sucrose, total soluble sugars, and starch) in plants grown to elevated CO_2_, according to life habits: Tree (**a**), Herbs (**b**), and Overall (**c**). The circles represent the percentage changes to elevated CO_2_. Error bars represent 95% confidence intervals. Study numbers for each variable are shown in parentheses.

The eCO_2_ increased plants' assimilation rate by 44% (Fig. 1; Table 1). Overall, trees + herbs responses in biomass showed an average increase of 20% in leaves, 41% in stems, and 43% in roots (Fig. 2; Table 1). The results in non-structural carbohydrates composed of glucose, fructose, sucrose, and starch present in the leaves of trees and herbs under CO_2_ are shown in Fig. 3. However, only total soluble sugars and starch content showed an increase of 7% and 47%, respectively (Fig. 3; Table 1).

**Table 1:** Meta-analysis with the percentage change of the biomass, photosynthesis, and biochemical variables measured in Trees and Herbs under elevated CO_2_. Observation numbers (k). The effect size values are represented as Log response rate (*LnRR*) and percentage. Average estimates with lower and upper Confidence Intervals (CI). Bold letters represent significant differences (p < 0.05).

		k	Mean (LnRR)	Lower CI	Upper CI	%change	p-value
Biomass variables							
Leaves	Trees	8	**1.07**	**0.23**	**1.92**	**194%**	**0.01**
	Herbs	7	0.04	− 0.03	0.31	4%	0.67
	Overall	15	**0.18**	**0.00**	**0.36**	**20%**	**< 0.05**
Stems	Trees	8	**1.23**	**0.39**	**2.08**	**245%**	**< 0.01**
	Herbs	5	**0.24**	**0.01**	**0.48**	**28%**	**0.03**
	Overall	13	**0.34**	**0.16**	**0.52**	**41%**	**< 0.01**
Roots	Trees	8	**1.25**	**0.40**	**2.09**	**250%**	**< 0.01**
	Herbs	2	**0.57**	**0.20**	**0.93**	**77%**	**< 0.01**
	Overall	10	**0.35**	**0.17**	**0.53**	**43%**	**< 0.01**
Grains	Trees	–	–	–	–	–	–
	Herbs	4	0.09	− 0.42	0.61	10%	0.71
	Overall	4	0.09	− 0.42	0.61	10%	0.71
Total	Trees	22	0.36	− 0.29	1.03	44%	0.27
	Herbs	15	0.14	− 0.03	0.31	15%	0.11
	Overall	37	0.21	− 0.07	0.35	24%	0.18
Photosynthesis variables							
Net CO_2_ assimilation (A)	Trees	28	**0.33**	**0.16**	**0.50**	**39%**	**< 0.05**
	Herbs	27	**0.42**	**0.21**	**0.63**	**52%**	**< 0.05**
	Overall	55	**0.36**	**0.23**	**0.49**	**44%**	**< 0.05**
Stomatal conductance (gs)	Trees	37	0.06	− 0.19	0.33	7%	0.6
	Herbs	18	− **0.50**	− **0.78**	− **0.22**	− **39%**	**< 0.05**
	Overall	55	− 0.19	− 0.39	0.00	− 17%	0.05
Dark respiration (Rd)	Trees	7	− **0.19**	− **0.36**	− **0.02**	− **17%**	**< 0.05**
	Herbs	3	0.09	− 0.17	0.37	10%	0.48
	Overall	10	− 0.13	− 0.31	0.04	− 12%	0.13
Foliar transpiration (E)	Trees	22	0.34	− 0.10	0.79	41%	0.13
	Herbs	19	− 0.29	− 0.77	0.17	− 25%	0.21
	Overall	41	0.04	− 0.30	0.38	4%	0.81
Water use efficiency (WUE)	Trees	20	− 0.16	− 0.76	0.42	− 15%	0.58
	Herbs	14	**1.02**	**0.33**	**1.70**	**117%**	**< 0.05**
	Overall	34	0.33	− 0.16	0.83	39%	0.19
Intercellular/ambient CO_2_ ratio (Ci/Ca)	Trees	9	− 0.03	− 0.12	0.05	− 3%	0.43
	Herbs	5	0.01	− 0.08	0.12	1%	0.71
	Overall	14	− 0.01	− 0.08	0.05	− 1%	0.68
Potential quantum efficiency of PSII (Fv/Fm)	Trees	12	0.07	0.01	0.16	7%	0.11
	Herbs	11	0.01	− 0.10	0.13	1%	0.78
	Overall	23	0.05	− 0.01	0.11	5%	0.14
Total chlorophyll (Chl total)	Trees	14	0.06	− 0.03	− 0.15	6%	0.22
	Herbs	12	0.05	− 0.05	0.15	5%	0.35
	Overall	26	0.05	− 0.01	0.12	5%	0.11
Maximum rate of electron transport (Jmax)	Trees	8	− 0.08	− 0.33	0.15	− 8%	0.48
	Herbs	2	0.31	− 0.15	0.79	37%	0.18
	Overall	10	− 0.00	− 0.21	0.21	0%	0.99
Maximum rate of Rubisco carboxylation (Vcmax)	Trees	8	− 0.22	− 0.48	0.04	− 19%	0.09
	Herbs	3	0.13	− 0.28	0.55	14%	0.53
	Overall	11	− 0.12	− 0.35	0.11	− 11%	0.31
Biochemical variables							
Glucose	Trees	8	0.10	− 0.04	0.25	10%	0.17
	Herbs	7	− 0.09	− 0.20	0.01	− 9%	0.07
	Overall	15	− 0.04	− 0.14	0.04	− 4%	0.28
Fructose	Trees	6	0.03	− 0.10	0.17	3%	0.6
	Herbs	7	**0.12**	**0.03**	**0.21**	**13%**	**< 0.01**
	Overall	13	0.06	− 0.05	0.14	7%	0.06
Sucrose	Trees	7	0.02	− 0.10	0.15	2%	0.6
	Herbs	6	**0.14**	**0.05**	**0.22**	**15%**	**< 0.01**
	Overall	13	**0.08**	**0.01**	**0.15**	**8%**	**0.01**
Total soluble sugars	Trees	6	− 0.00	− 0.15	0.13	0%	0.89
	Herbs	4	**0.13**	**0.04**	**0.21**	**14%**	**< 0.01**
	Overall	10	**0.07**	**0.00**	**0.14**	**7%**	**0.04**
Starch	Trees	7	**0.47**	**0.08**	**0.87**	**61%**	**0.01**
	Herbs	14	0.29	− 0.09	0.69	34%	0.14
	Overall	21	**0.38**	**0.11**	**0.65**	**47%**	**< 0.01**
Proteins	Trees	4	0.10	− 0.39	0.61	11%	0.67
	Herbs	3	0.03	− 0.04	0.46	3%	0.88
	Overall	7	0.06	− 0.23	0.35	6%	0.68

### Elevated CO_2_ effect in trees and herbs according to life habits

**Figure 4:**
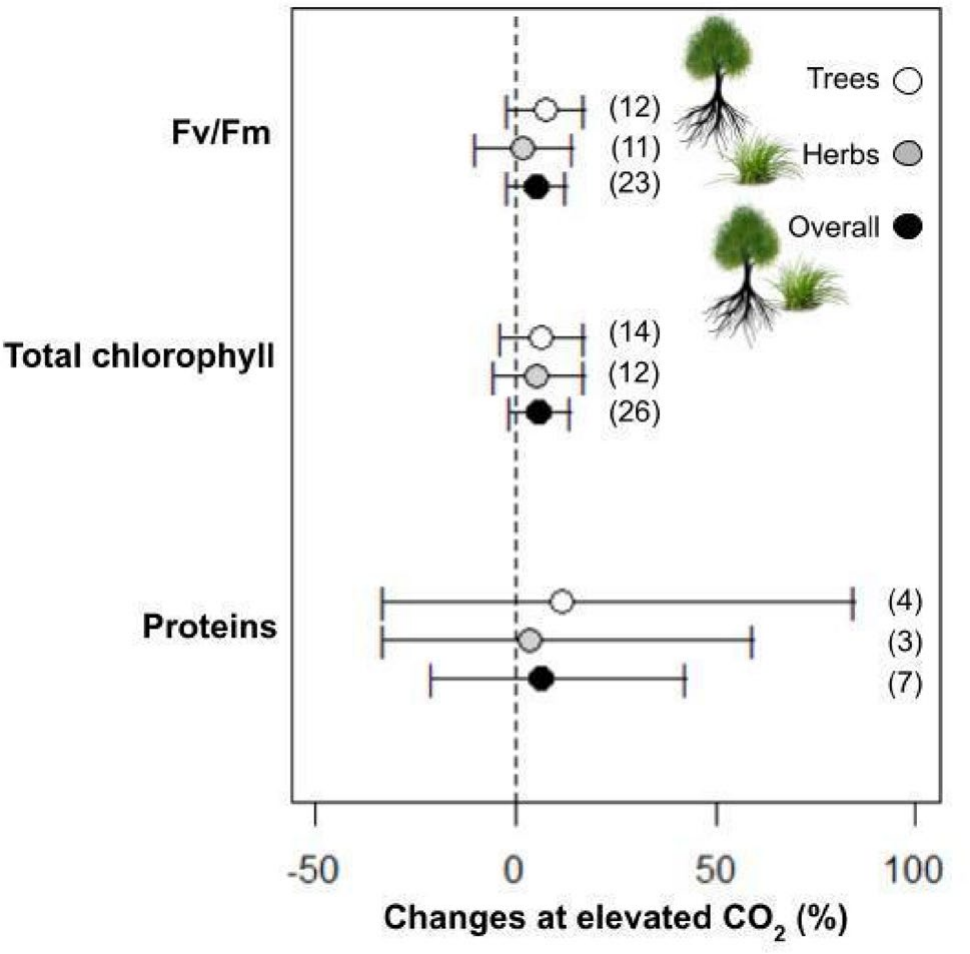
Responses of potential quantum efficiency of photosystem II (Fv/Fm), total chlorophyll content, and proteins in plants grown in elevated CO_2_, according to life habits: Trees (white), Herbs (gray), and Overall (black). The circles represent the percentage change in elevated CO_2_. Error bars represent 95% confidence intervals. Study numbers for each variable are shown in parentheses.

**Figure 5:**
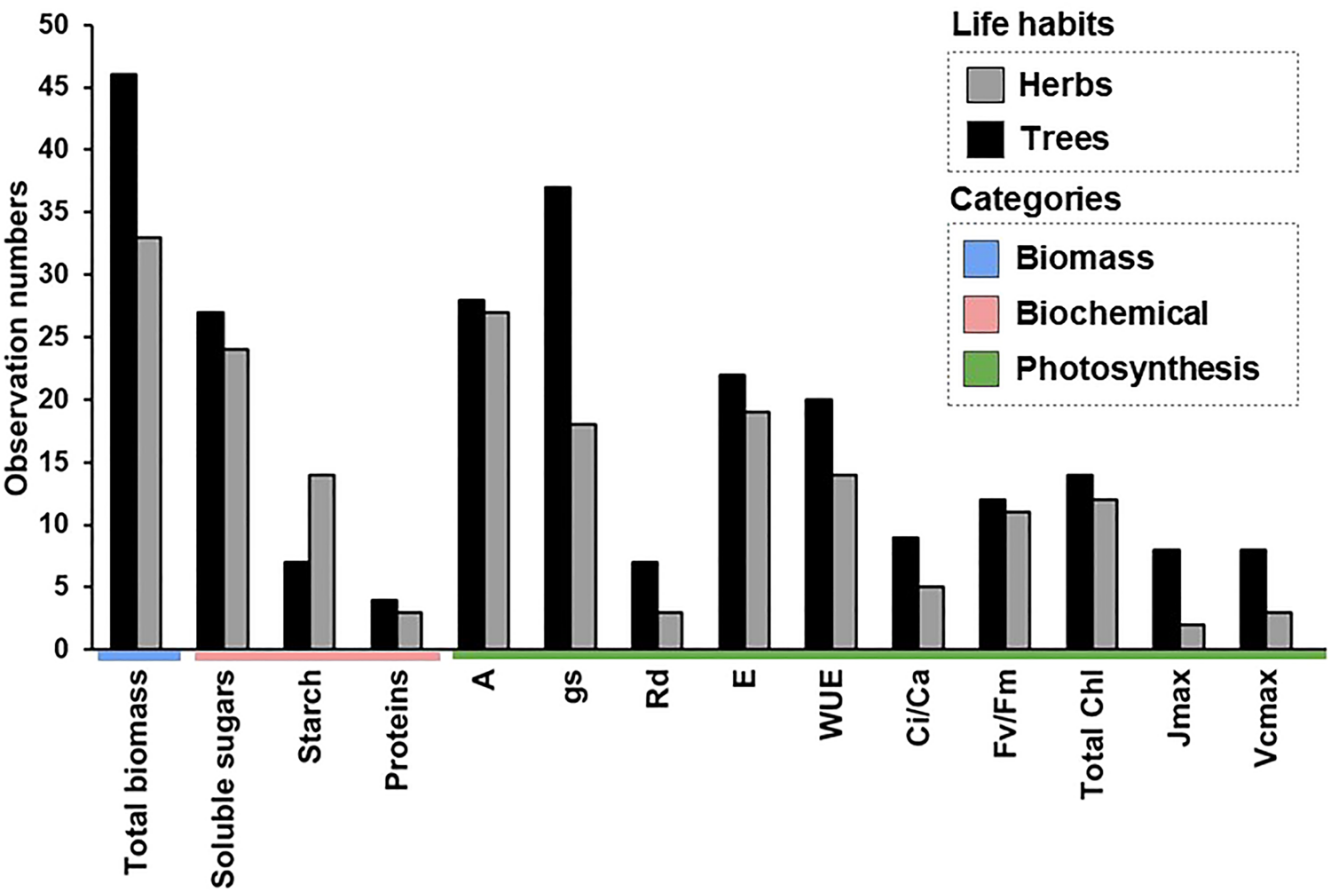
Observation numbers from the literature extracted were divided into biomass, biochemical, and photosynthesis components according to life habits: Trees (black) and Herbs (gray) in experiments with elevated CO_2_. The variables correspond to total biomass, total soluble sugars, starch, proteins, net CO_2_ assimilation (A), stomatal conductance (gs), foliar transpiration foliar (E), water use efficiency (WUE), dark respiration (Rd), intercellular/ambient CO_2_ ratio (Ci/Ca), the potential quantum efficiency of PSII (Fv/Fm), total chlorophyll (total Chl) maximum Rubisco carboxylation rate (Vc_max_), and maximum electron transport rate (J_max_).

The life habits were essential to distinguish responses in total biomass, stomatal conductance (*gs*), transpiration foliar (*E*), water use efficiency (*WUE*), and maximum rate of electron transport (*J*_*max*_) (Table 2). The biomass increase is different per organ between trees and herbs under eCO_2_. The biomass increased more in trees than in the herbs category, being higher on leaves (194%), stems (245%), and roots (250%) (Fig. 2; Table 1). In herbs, the biomass increased by 28% and 77% in stems and roots, respectively. Furthermore, changes in the biomass of leaves were not significant in herbs (Fig. 2; Table 1). The grain biomasses were only measured in herbs, which had no alteration in plants cultivated under eCO_2_ (Fig. 2). Starch increased by 61% in trees, while in the herbs, the fructose, sucrose, and soluble sugars increased by 13%, 15%, and 14%, respectively (Fig. 3; Table 1). When trees and herbs were analyzed separately, the assimilation increased by 39% and 52%, respectively (Fig. 3; Table 1). Stomatal conductance negatively affected herbs (p = 0.001; Table 2; Fig. 1). The reduction of *gs* (39%) in herbs increased *WUE* (117%) (Fig. 3). Thus, the photosynthesis parameters *WUE*, *E*, and *J*_*max*_ differed among herbs and trees at eCO_2_ (Fig. 1; Table 2). These results may reflect a tendency for the opposite effects of these variables in trees and herbs (Fig. 1; Table 1). On the other hand, the trees displayed no significant effect in *gs*, *WUE*, and *E* at eCO_2_ (Fig. 1; Table 1). Under eCO_2_, trees significantly reduced dark respiration (17%). Furthermore, Ci/Ca, Jmax, Vcmax, Fv/Fm, and total Chl in trees and herbs under eCO_2_ did not change under eCO_2_ (Figs. 1, 4). The lack of effect could reflect the small number of observations in those variables (Fig. 5), which calls for more studies to provide consistent analysis for these variables.

**Table 2:** Meta-analyses result in different variables according to life habits: Trees and Herbs, publication bias, and heterogeneity. Bold letters represent significant differences between Trees and Herbs p < 0.05. For data from the column in publication bias, the p-value < 0.05 does not indicate publication bias. For heterogeneity, analyses were considered I^2^ ≤ 25 low, I^2^ > 25 to 75 moderate, and I^2^ > 75 high heterogeneity.

	Life habits	Publication bias	Heterogeneity (%)
Total biomass	**< 0.01**	**0.01**	95
Net CO_2_ assimilation (A)	0.89	0.92	99
Stomatal conductance (gs)	**< 0.01**	**< 0.01**	96
Dark respiration (Rd)	0.07	0.08	92
Foliar transpiration (E)	**0.05**	0.14	98
Water use efficiency (WUE)	**< 0.01**	0.11	90
Intercellular/ambient CO_2_ ratio (Ci/Ca)	0.43	0.52	81
Maximum rate of electron transport (J_max_)	**0.05**	**< 0.01**	88
Maximum rate of Rubisco carboxylation (Vc_max_)	0.16	0.31	63
Potential quantum efficiency of PSII (Fv/Fm)	0.46	0.75	13
Total chlorophyll (Chl total)	0.89	0.24	93
Total soluble sugars	0.29	0.33	89
Starch	0.23	**0.01**	91
Proteins	0.74	0.74	99

### Heterogeneity and publication bias analysis

Heterogeneity (I^2^) analysis in the analytical models was used to evaluate the variation in results among observations. The high heterogeneity indicates variation in the effect of eCO_2_ among observations. The heterogeneity was high (I^2^ > 75) for total biomass, *A, gs, Rd, E, WUE, Ci/Ca, J*_*max*_*, total chl,* starch, and proteins (Table 2). High heterogeneity shows that external factors may influence the variation of the estimated effects among observations. The *Vc*_*max*_ showed moderate heterogeneity, and the *Fv/Fm* had low heterogeneity (Table 2). These results demonstrate less variation among observations in *Vc*_*max*_ and Fv/Fm variables.

No publication bias was found for net CO_2_ assimilation, dark respiration, foliar transpiration, water use efficiency, intercellular/ambient CO_2_ rate, maximum carboxylation rate, the potential quantum efficiency of PSII, total chlorophyll, and total soluble sugars (Table 2). However, the Egger test identified publication bias for biomass, *gs*, *J*_*max*_, and starch (Table 2).

## Discussion

**Figure 6:**
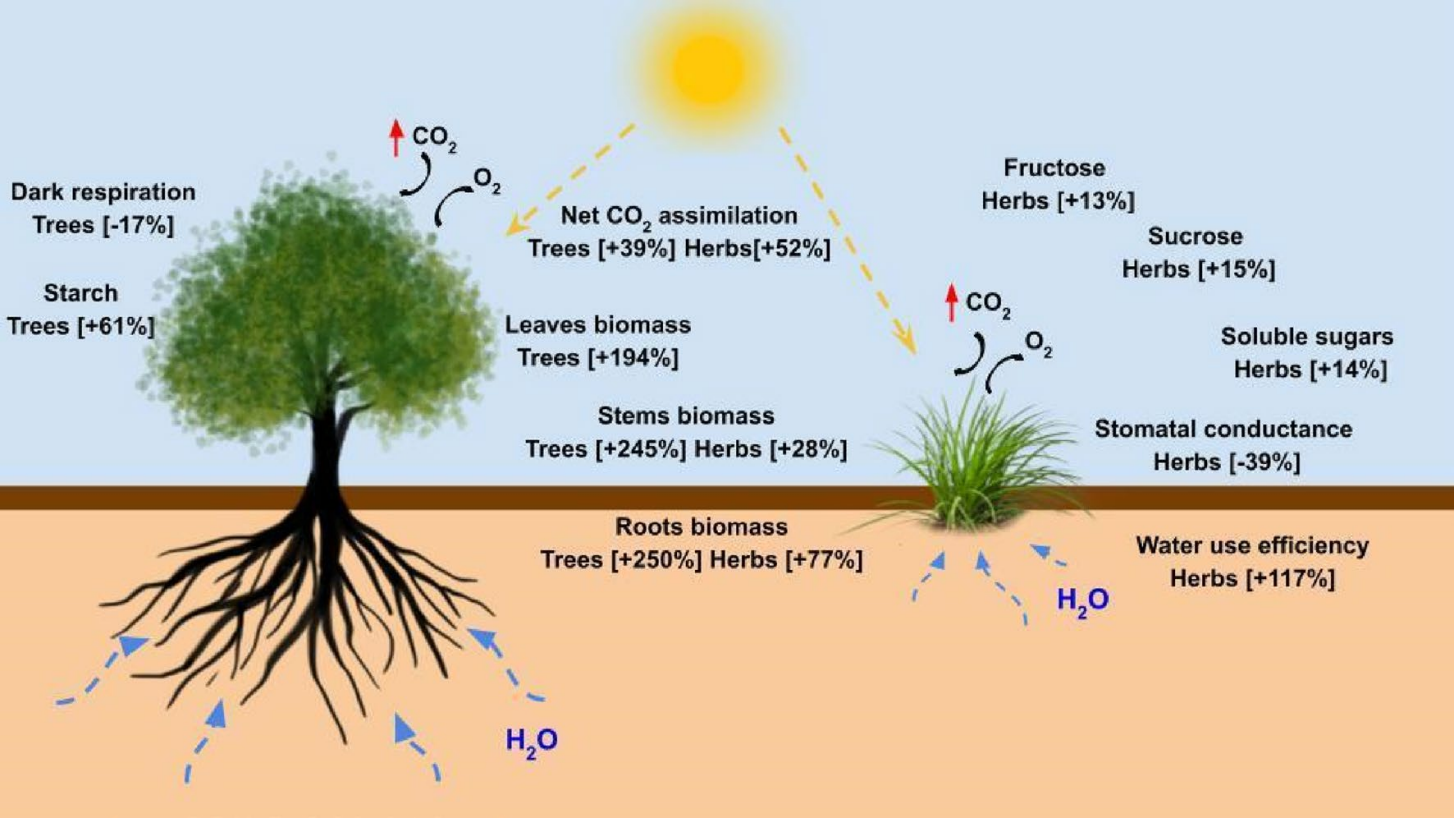
Tropical climate trees and herbs responses to elevated CO_2_.

Plants can be used to capture carbon to delay the effects of climate change through photosynthesis, which assimilates carbon in the form of CO_2_ and accumulates it into the plant's biomass. Thus, higher carbon availability is expected to generate changes in these processes and intensify plant growth^15,47^. In the meta-analysis presented in this work, data from species planted as crops and native species to the neotropics were examined. We confirmed previous literature observations regarding the physiology of temperate species, showing that several neotropical ones alter their photosynthesis parameters, biomass accumulation, and sugars (biochemicals) under eCO_2_ (Fig. 6). The elevated CO_2_ in plants through photosynthesis is directly connected to their growth and productivity^48^. In addition, elevated CO_2_ stimulated photosynthetic assimilation in neotropical herbs, improving *WUE* due to stomata closure and conductance reduction (Fig. 1). This behavior corroborates evidence reported in other meta-analyses^12,28^, except that neotropical trees did not alter the stomatal conductance responses as happens in temperate trees^13^.

Stomatal conductance (*gs*) and assimilation rates control the intercellular/ambient CO_2_ ratio, which dictates the internal carbon allocation in plants^49^. Elevated CO_2_ increases the concentration of intracellular CO_2_ in leaves^38^, but to continue the assimilation, the mesophyll CO_2_ needs to display lower concentrations than the atmospheric partial pressure of CO_2_^50^. This regulation is performed by the closure and opening of the stomata, which leads to a decrease in stomatal conductance^38,51^.

It has been reported that European forests grown in eCO_2_ decreased *J*_*max*_ and *Vc*_*max*_ by 10%^52^. The authors attributed this decrease to the limiting levels of nitrogen in leaves. The Neotropical species examined in the present work did not decrease *J*_*max*_ and *Vc*_*max*_ changes (Fig. 1), possibly indicating that the leaf nitrogen status in the experiments used for this meta-analysis was not limited. According to Bonan et al.^53^, the *Vc*_*max*_ parameter displays relevant implications for large-scale modeling. Carbon flux models show that simulated photosynthetic rates are particularly susceptible to *Vc*_*max*_ and *J*_*max*_, with the former being pointed out by Bonan et al.^53^ as a model-dependent parameter. Therefore, accuracy in these parameters is critical for a more effective prediction and modeling by the global panels.

The sugars produced during photosynthesis can be metabolized for maintenance and developmental processes. Catabolism of sugars leads to the consumption of ATP by respiration, which may increase or decrease, depending on the species, when plants are exposed to unfavorable conditions^54^. When neotropical plant species were subjected to elevated CO_2_ during growth, they displayed a decrease in dark respiration (*Rd*) (see Overall in Fig. 1), which is expected to increase the efficiency of the net productivity of carbon gain^55,56^. Thus, the efficiency of the carbon metabolism increases under eCO_2_. The decrease in *Rd* may be associated with the higher concentrations of foliar starch found in plants grown under eCO_2_ analyzed in Overall (Table 1). The same pattern of reduction of *Rd* was observed for temperate trees^12^. However, no meta-analysis has been performed considering sugar metabolism and photosynthesis, so temperate and neotropical species could not be directly compared via meta-analysis.

An explanation for the higher accumulation of starch in leaves of neotropical species growing under eCO_2_ is that the photosynthetic assimilation rate can exceed the growth capacity, leading to the accumulation of non-structural carbohydrates^19,57,58^. We found that starch increase (47%) represents the primary non-structural carbohydrate in plant leaves under eCO_2_ (Fig. 2).

The increased starch levels in eCO_2_ are usually the main element responsible for increasing the content of total non-structural carbohydrates^59^. Starch is composed of insoluble and long-term storage polysaccharides (amylose and amylopectin) that are not readily available to participate in plant metabolic processes^60^ but can be used to increase biomass in leaves, stems, and roots, as observed in this meta-analysis (Fig. 2). The carbohydrates synthesized in leaves from extra CO_2_ supply were translocated into tree stems (Fig. 2), suggesting that the reserve biomass is driven to this organ, boosting secondary growth^61^. Furthermore, stimulation of photosynthesis with eCO_2_ had a response in the biomass increase different in the development of organs and plant seed mass^62^. Li et al.^63^ synthesized 71 tree species and data of a more significant increase in starch than soluble sugars in leaves under eCO_2_.

The results obtained in this work show that the responses of neotropical plant species to eCO_2_ are consistent with those on the global scale (temperate climates mainly), suggesting that the predictions made by models of climate change would answer similarly to temperate and neotropical species^13,47,52^. However, in Brazil, relatively few experiments were carried out with eCO_2_ in plants from the biomes Pantanal, Caatinga, Cerrado, Amazon, and the Pampas, the latter in a temperate region (Table 3). Thus, more profound exploration should provide relevant information on how different biomes could answer to eCO_2_ and climate change^64,65^. Also, establishing long-term experiments to test the effect of eCO_2_ on plants over time in Brazil is needed once a significant portion of the neotropical plants is located there. This would allow an understanding of the physiological responses to climate change^66^.

**Table 3:** Species found in a literature search with plants grown at different CO_2_ atmospheric concentrations (ambient CO_2_ = aCO_2_ and elevated CO_2_ = eCO_2_), classified according to life habits: Tree and Herbs. *OTC* Open top chambers, *FACE* Free Air Carbon Enrichment, and *GC* Glasshouse, *ppm* parts per million.

Species	aCO_2_	eCO_2_	Functional group	Native/exotic/cultivated	Experiment	Reference
*Acrocomia aculeata*	400	700	Tree	Native	OTC	Rosa et al. 2019
*Alchornea glandulosa*	400	800	Tree	Native	OTC	Fauset et al. 2019
*Anacardium occidentale*	380	720	Tree	Native/cultivated	GC	Souza, 2012
*Anacardium occidentale*	380	760	Tree	Native/cultivated	GC	Souza et al. 2019
*Anadenanthera peregrina*	430	700	Tree	Native	OTC	Melo, 2020
*Baccharis dracunculifolia*	360	720	Tree	Exotic/cultivated	OTC	Sá et al. 2014
*Carapa surinamensis*	400	700	Tree	Native	GC	Oliveira, 2016
*Carapa surinamensis*	350	1000	Tree	Native	OTC	Oliveira, 2017
*Cariniana legalis*	380	740	Tree	Native	OTC	Martinez et al. 2008
*Cariniana legalis*	380	760	Tree	Native	OTC	Oliveira et al. 2012
*Coffea arabica*	400	700	Tree	Exotic/cultivated	OTC	Avila et al. 2020
*Coffea arabica*	400	550	Tree	Exotic/cultivated	FACE	Bianconi, 2014
*Coffea arabica*	390	550	Tree	Exotic/cultivated	FACE	Ghini et al. 2015
*Coffea arabica*	380	740	Tree	Exotic/cultivated	OTC	Marçal et al. 2021
*Coffea arabica*	380	700	Tree	Exotic/cultivated	GC	Martins et al. 2016
*Coffea arabica*	390	590	Tree	Exotic/cultivated	FACE	Rakocevic et al. 2016
*Coffea arabica*	390	590	Tree	Exotic/cultivated	FACE	Rakocevic et al. 2018
*Coffea arabica*	380	700	Tree	Exotic/cultivated	GC	Ramalho et al. 2018
*Coffea arabica*	380	760	Tree	Exotic/cultivated	OTC	Reis, 2015
*Coffea arabica*	380	700	Tree	Exotic/cultivated	GC	Rodrigues et al. 2016
*Coffea arabica*	400	760	Tree	Exotic/cultivated	OTC	Sanches et al. 2017
*Coffea arabica*	380	700	Tree	Exotic/cultivated	GC	Semedo et al. 2021
*Coffea canephora*	380	700	Tree	Exotic/cultivated	GC	Martins et al. 2016
*Coffea canephora*	380	700	Tree	Exotic/cultivated	GC	Rodrigues et al. 2016
*Coffea canephora*	380	700	Tree	Exotic/cultivated	GC	Semedo et al. 2021
*Coffea sp.*	390	550	Tree	Exotic/cultivated	FACE	DaMatta et al. 2015
*Croton urucurana*	380	740	Tree	Native	OTC	Martinez et al. 2008
*Croton urucurana*	380	760	Tree	Native	OTC	Oliveira et al. 2012
*Dalbergia nigra*	360	720	Tree	Native	OTC	Godoy, 2007
*Enterolobium contortisiliquum*	380	700	Tree	Native	OTC	Melo, 2015
*Enterolobium contortisiliquum*	400	700	Tree	Native	OTC	Melo et al. 2018
*Eucalyptus sp*	380	700	Tree	Exotic/cultivated	FACE	Fontes, 2017
*Eucalyptus sp.*	400	760	Tree	Exotic/cultivated	OTC	Baesso, 2017
*Euterpe oleracea*	380	760	Tree	Native/cultivated	OTC	Mortari, 2015
*Hymenaea courbaril*	360	720	Tree	Native	OTC	Godoy, 2007
*Hymenaea courbaril*	360	720	Tree	Native	OTC	Aidar et al. 2002
*Hymenaea courbaril*	360	720	Tree	Native	OTC	Costa, 2004
*Hymenaea courbaril*	370	720	Tree	Native	OTC	Machado, 2007
*Hymenaea courbaril*	380	760	Tree	Native	OTC	Mayorga, 2010
*Hymenaea stigonocarpa*	370	720	Tree	Native	OTC	Machado, 2007
*Hymenaea stigonocarpa*	390	1000	Tree	Native	OTC	Maia, 2016
*Hymenaea stigonocarpa*	380	700	Tree	Native	OTC	Melo, 2015
*Hymenaea stigonocarpa*	430	700	Tree	Native	OTC	Melo, 2020
*Hymenaea stigonocarpa*	400	700	Tree	Native	OTC	Souza et al.2018
*Lafoensia pacari*	430	700	Tree	Native	OTC	Souza et al. 2019
*Piptadenia gonoacantha*	360	720	Tree	Native	OTC	Godoy, 2007
*Psidium guajava*	390	780	Tree	Native/cultivated	OTC	Rezende et al. 2015
*Schizolobium parahyba*	360	720	Tree	Native	OTC	Godoy, 2007
*Schizolobium parahyba*	360	720	Tree	Native	OTC	Godoy, 2007
*Senna alata*	380	700	Tree	Native	OTC	Marabesi, 2007
*Senna reticulata*	380	760	Tree	Native	OTC	Arenque-Musa, 2010
*Senna reticulata*	380	760	Tree	Native	OTC	Arenque-Musa et al. 2014
*Senna reticulata*	400	800	Tree	Native	OTC	Arenque-Musa, 2014
*Senna reticulata*	380	760	Tree	Native	OTC	Grandis, 2010
*Sesbania virgata*	360	720	Tree	Native	OTC	Godoy, 2007
*Sesbania virgata*	360	720	Tree	Native	OTC	Godoy, 2007
*Solanum lycocarpum*	400	700	Tree	Exotic/cultivated	OTC	Souza et al. 2018
*Stryphnodendron adstringens*	430	700	Tree	Native	OTC	Melo, 2020
*Stryphnodendron polyphyllum*	430	700	Tree	Native	OTC	Melo, 2020
*Tabebuia aurea*	430	700	Tree	Native	OTC	Melo, 2020
*Tabebuia aurea*	400	700	Tree	Native	OTC	Souza et al. 2018
*Brachiaria decumbens*	390	550	Herbaceous	Exotic/cultivated	FACE	Abdalla, 2018
*Chrysolaena obovata*	380	760	Herbaceous	Native	OTC	Oliveira et al. 2016
*Glycine max*	360	720	Herbaceous	Exotic/cultivated	OTC	Braga et al. 2006
*Glycine max*	360	720	Herbaceous	Exotic/cultivated	OTC	Costa, 2003
*Glycine max*	380	760	Herbaceous	Exotic/cultivated	OTC	Kretzschmar, 2007
*Glycine max*	380	760	Herbaceous	Exotic/cultivated	OTC	Kretzschmar et al. 2009
*Glycine max*	360	720	Herbaceous	Exotic/cultivated	OTC	Lobo, 2003
*Melinis minutiflora*	380	700	Herbaceous	Exotic/cultivated	OTC	Melo, 2015
*Melinis minutiflora*	350	1000	Herbaceous	Exotic/cultivated	OTC	Oliveira, 2017
*Oryza sativa*	400	700	Herbaceous	Exotic/cultivated	OTC	Barbosa, 2019
*Oryza sativa*	400	700	Herbaceous	Exotic/cultivated	OTC	Dorneles et al. 2020
*Panicum maximum*	390	600	Herbaceous	Exotic/cultivated	FACE	Approbato, 2015
*Panicum maximum*	400	600	Herbaceous	Exotic/cultivated	FACE	Bortolin, 2016
*Panicum maximum*	400	600	Herbaceous	Exotic/cultivated	FACE	Britto, 2016
*Panicum maximum*	400	600	Herbaceous	Exotic/cultivated	FACE	Habermann et al. 2019
*Panicum maximum*	400	600	Herbaceous	Exotic/cultivated	FACE	*Habermann *et al*. 2020*
*Panicum maximum*	385	600	Herbaceous	Exotic/cultivated	FACE	Oliveira et al. 2020
*Phaseolus vulgaris*	380	700	Herbaceous	Exotic/cultivated	OTC	Silva, 2010
*Saccharum sp.*	370	720	Herbaceous	Exotic/cultivated	OTC	De Souza, 2007
*Saccharum sp.*	370	720	Herbaceous	Exotic/cultivated	OTC	De Souza et al. 2008
*Saccharum sp.*	390	750	Herbaceous	Exotic/cultivated	OTC	De Souza, 2011
*Solanum curtilobum*	360	720	Herbaceous	Exotic/cultivated	OTC	Olivo et. 2002
*Solanum lycopersicum*	400	750	Herbaceous	Exotic/cultivated	OTC	Brito, 2016
*Solanum lycopersicum*	400	750	Herbaceous	Exotic/cultivated	OTC	Pimenta, 2017
*Solanum tuberosum*	360	720	Herbaceous	Exotic/cultivated	OTC	Olivo et. 2002
*Stylosanthes capitata*	400	600	Herbaceous	Native	FACE	Habermann et al. 2019
*Urochloa brizantha*	360	550	Herbaceous	Native	OTC	Faria et al. 2015
*Vernonia herbacea*	380	720	Herbaceous	Native	OTC	Oliveira, 2007
*Vernonia herbacea*	380	760	Herbaceous	Native	OTC	Oliveira et al. 2010
*Vernonia herbacea*	360	760	Herbaceous	Native	OTC	Oliveira, 2012
*Viguiera discolor*	380	760	Herbaceous	Native	OTC	Oliveira et al. 2013
*Zea mays*	380	700	Herbaceous	Exotic/cultivated	OTC	Silva, 2010

Native plants in neotropical regions have evolved to adapt to their specific environmental conditions, including CO_2_ levels. Elevated CO_2_ can positively affect native plants by increasing photosynthesis, promoting plant growth, increasing carbon sequestration, and potentially acting as a CO_2_ sink^16^. In contrast, plants grown in neotropical regions are often grown for agricultural purposes. They may have different responses to eCO_2_ compared to native plants, although this hypothesis needs to be checked in further studies with more species. Cultivated plants can exhibit increased photosynthetic rates and grow under elevated CO_2_^35^. This can benefit crop productivity and potentially increase carbon sequestration in farming systems^67^. However, the response of cultivated plants to elevated CO_2_ may vary depending on factors such as plant species, nutrient availability, management practices, and genetic improvement techniques^68^. Therefore, it is important to note that the potential of native and cultivated plants to act as CO_2_ sources or sinks is influenced by several factors. These include the specific plant species, their physiological characteristics, duration of exposure to elevated CO_2_, and general ecosystem dynamics. To understand the potential of native and cultivated plants in neotropical regions as sources or sinks of CO_2_, more research is needed.

Figure 6 summarizes the responses of the neotropical species analyzed in this work. Temperate and neotropical species respond similarly to eCO_2_, which is likely to reflect directly in the consistency of modeling regarding the adjustment of parameters. Trees and herbs display different responses. The trees studied are primarily young and, therefore, rapidly growing. As they are not yet at the reproductive stage, young trees tend to allocate carbon—from increased photosynthetic rates and lower respiration in the dark—to organ development, significantly increasing leaves, roots, and stem biomasses. As growth rates are limited in comparison with the growth capacity of most herbs, more starch is accumulated in trees, denoting a tight control of carbon metabolism through carbohydrate storage. Herbs, mainly crop plants, reached reproductive maturity during the experiments. Their strategy to respond to eCO_2_ involved a drastic increase in water use efficiency, controlled by stomatal conductance. In addition, the plants tend to display more soluble sugars, probably with a transient accumulation of carbon primarily stored in seeds.

## Conclusion

The responses of species native or cultivated in the neotropics to eCO_2_ can be attributed to contrasting growth strategies and physiological features of trees and herbs. Trees display greater carbon sink capacity and can allocate more resources for growth and storage. The higher rates of photosynthesis in response to eCO_2_ (39%) led to greater starch storage (61%) and a more significant biomass accumulation in tree organs (Table 1). This behavior may be attributed to the tree’s long lifespan and ability to allocate resources for growth and storage.

In contrast, herbs, which display shorter lifespans, prioritize rapid growth and reproduction and tend to allocate resources that would support higher water use efficiency (117%) due to decreased stomatal conductance (− 39%) under conditions of eCO_2_. Herbs responded differently, increasing net CO_2_ assimilation (52%) and soluble sugars such as sucrose and fructose (14%, 15%, and 13%). Understanding these responses would be crucial to predicting the impacts of increased CO_2_ levels on different types of plants in the face of eCO_2_ increases.

Finally, it is essential to note that eCO_2_ alone does not represent the complete response of plants to climate change. Combinations of eCO_2_ with stresses of temperature and water will be necessary to assess the systemic response of plants to global climate change. Thus, more experiments are needed using these parameters that, together with modeling work, could help understand how the neotropics, with their rather large proportion of world biodiversity, will respond to climate change in this century.

## Materials and methods

### Data collection

For data collection, a systematic review was performed. A systematic review is a technique that selects primary studies on a given subject^69^. For the elaboration of the systematic review, it is necessary to identify and describe the steps taken to study selection and data extraction. These steps must follow a protocol that can be consulted and reproducible^69^. The flowchart with steps for data collection is shown in Supplementary Fig. 1. Literature search for the data collection on the effect of the elevated CO_2_ on plants was performed in three databases: W*eb of Science, Scielo,* and *Brazilian Digital Library of Theses and Dissertations* (https://bdtd.ibict.br)^70–72^. For each database, a combination of keywords was used (Supplementary Table 1) that recovered 2096 works on the eCO_2_. In addition, 35 studies were manually included from leading Brazilian researchers by Lattes search (https://lattes.cnpq.br)^72^. Lattes is a Brazilian platform for integrating Curriculum, Research Groups, and Institution databases into a single information system^72^. The search resulted in a total of 2127 analyzed works in the systematic review (Supplementary Fig. 1). A database was assembled with 68 studies published before October 1st, 2021 (see Table 3; Supplementary Fig. 1). The included works were: (a) studies on Brazilian manipulative experimentation, reporting results from both the treatment groups (eCO_2_) and the control groups (ambient CO_2_ = aCO_2_); (b) studies on trees or herbs; and (c) studies with the mean, sample size, and standard deviation of error of the selected variables. The data from articles were grouped as trees and herbs on 28 and 16 species, respectively (Table 3). The collected data were extracted in three theoretical categories: growth (biomass), biochemical (total soluble sugars, starch, and proteins), and photosynthesis-related parameters [net CO_2_ assimilation (*A*), stomatal conductance (*gs*), transpiration foliar (*E*), water use efficiency (*WUE*), dark respiration (*Rd*), intercellular/ambient CO_2_ ratio (*Ci/Ca*), the potential quantum efficiency of PSII (*Fv/Fm*), total chlorophyll content (*Chl*), maximum carboxylation rate (*Vc*_*max*_), and maximum rate of electron transport (*J*_*max*_)]. The biomass data were collected from total biomass or biomass per plant organ. Each biomass result per organ was considered a biomass observation. Each soluble sugar (glucose, fructose, sucrose, raffinose, and myoinositol) was considered an observation for the biochemical category. A dataset contemplated a total of 437 observations. In general, the duration of the studies was 90 days. The average high CO_2_ concentration was from ~ 400 to ~ 800 ppm. Fifty studies were performed in Open Top Chambers (OTC), 13 in Free Air CO_2_ Enrichment (FACE), and 7 in Glasshouse (GC). The most frequently studied species among trees was *Coffea arabica*, with 12 different studies. On the other hand, among herbs was *Panicum maximum* with six different studies. Fourteen variables were analyzed [*A, gs, E, WUE, Rd, Ci/Ca, biomass, total soluble sugars, starch, proteins, Fv/Fm, total Chl, Vc*_*max*_*, J*_*max*_] (Fig. 5). The most frequent variables were biomass (79), with 46 observations for trees and 33 for herbs (Fig. 5). From the total species analyzed, 30% represent cultivated ones. Among the trees, 21% are cultivated, and 79% are native species. Among herbs, 33% are native, and 67% are cultivated. The experiments were considered unstressed unless the author had identified some stress factor. In the case of stress treatments, data from the control treatments were used. Most of the works had an average duration of experimentation of 90 days. The plants were grown in pots. Plants that received fertilizer treatment were not included in this analysis. The plants were watered regularly and exposed to natural light.

Observations of each study at the end of the experiment were grouped, and there was no categorization by experiment period. There was also the group for the elevated CO_2_ levels of the different studies. Curtis and Wang^13^ examines each subgroup for categorical divisions such as pot size and exposure time. However, a meta-analysis by these authors did not find significant differences among the groups by pot size and experiment time. This is an example that, throughout all studies, suggests significant differences in the response of plants under the CO_2_ environment and, however, not among those grown in different pot sizes or experiment duration.

Mean values, standard deviation/error, and sample size under eCO_2_ and aCO_2_ were collected for each observation. WebPlotDigitizer v4.1^73^ was used to obtain the numerical data from the figures. For works that showed only the standard error value, the following equation was used: (SD = SE × √n) (n is the sample size, SE is the standard error, and SD is the standard deviation)^74^. Data from temporal experiments were considered only the last harvest to represent the maximum exposure of these plants to eCO_2_ cultivation.

### Meta-analysis

Meta-analysis assessed plant responses to eCO_2_ in growth, biochemical composition, and photosynthesis categories. To evaluate the relative changes of these responses between treatment (eCO_2_) versus control (aCO_2_), it was applied the logarithmic response ratio ln (RR), calculated as the size effect, where X̅t is the mean of the experimental/treatment group, and X̅c is the mean of the control group^68^. The natural log of the response ratio (lnRR = X̅t/X̅c) was used and is reported as the mean percentage change [(lnRR − 1) × 100]^75^. Values of lnRR higher than zero indicate that the eCO_2_ effect increases, while negative values indicate that the eCO_2_ effect decreases concerning aCO_2_. A hierarchical mixed-effects model was used to estimate the mean and 95% confidence interval (CI) of the lnRR for each type of response variable. If the 95% CI of a response variable overlaps zero, the lnRR of the treatment is not significantly different from the control^76^. The effect was reported as a percentage change from the control: ((e^lnRR^ − 1) × 100). In addition, life habits were used as a fixed predictor variable while the study and species were considered random variables to control for the lack of independence of observations from the same study or/and carried out with the same plant species^77,78^. Furthermore, heterogeneity (I^2^) was tested to verify the variation in results between studies^77,79^. The Egger regression test was used to identify publication bias^80,81^. Bias analyses for the multilevel models were conducted with meta-analytic residuals^77^. Analyzes were performed using the package "*metafor*"^82^, and the graphics were generated using the package "*ggplot2*"^78^, both in R version program 3.6.0^83^.

### Supplementary Information

Supplementary Information 1.

Supplementary Information 2.

Supplementary Information 3.

## Data Availability

All data generated or analyzed during this study are included in this published article as supplementary information file (excel) named “Supplementary Table 2”.
