# Supplementary material for: Meta-analysis of the responses of tree and herb to elevated CO2 in Brazil
Source: Sci Rep. 2023 Sep 22;13:15832. doi: 10.1038/s41598-023-40783-5 (PMC10517018; doi:10.1038/s41598-023-40783-5)
Supplement: Supplementary file 1 — Supplementary Information 1. [file 41598_2023_40783_MOESM1_ESM.docx]

**Supplemental Figure and Table 2**


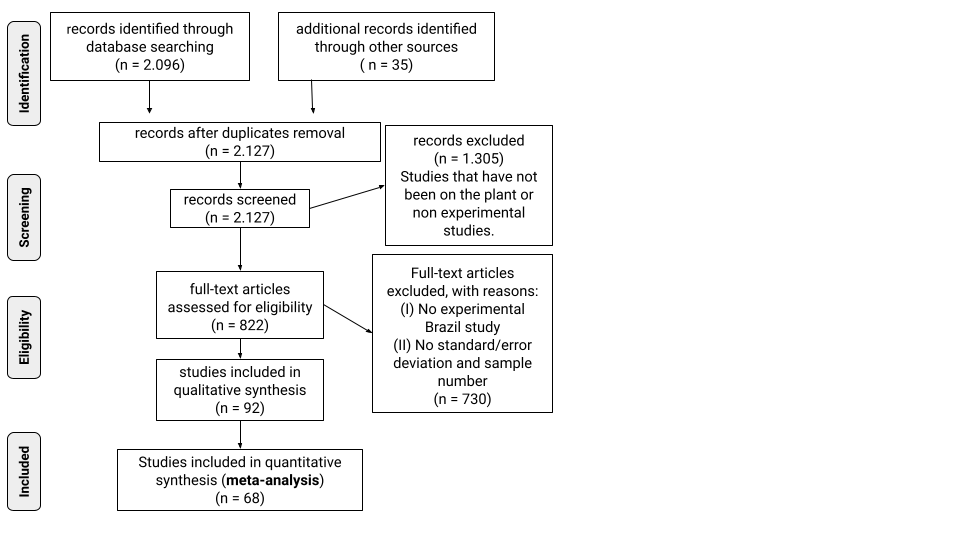


**Supplemental Figure 1**. Flowchart showing the steps of the systematic review, modified from (Liberati et al., 2009). Numbers in parentheses represent the works included/excluded in each step of the systematic review.

**Supplemental Table 1.** Strategies used in each database with different keyword combinations. Asterisks represent any letters that were used as word complements. This survey was last conducted on October 1st, 2021, and returned 2,096 studies analyzed for eligibility for the meta-analysis. BDTD (*Brazilian Digital Library of Theses and Dissertations)*


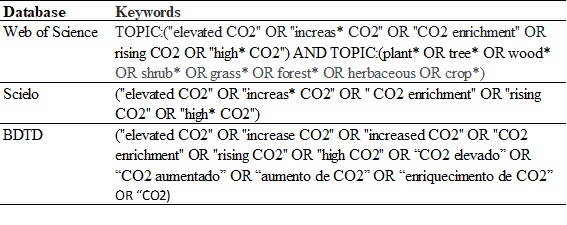


**Supplemental Table 2**. All data used in this meta-analysis (supplied as an excel file)
