# Supplementary material for: Meta-analysis of the responses of tree and herb to elevated CO2 in Brazil
Source: Sci Rep. 2023 Sep 22;13:15832. doi: 10.1038/s41598-023-40783-5 (PMC10517018; doi:10.1038/s41598-023-40783-5)
Supplement: Supplementary file 3 — Supplementary Information 3. [file 41598_2023_40783_MOESM3_ESM.docx]

**References of the works included in the meta-analysis (See Table 3)**

Abdalla, F. A. L. Quantificação e caracterização da biomassa produzida em condições de simulação de aumento da concentração de CO_2_ atmosférico no sistema agropastoril. Tese de Doutorado, Centro de Energia Nuclear na Agricultura, Universidade de São Paulo, Piracicaba.https:10.11606/T.64.2018.tde-12112018-153146 (2018).

Aidar, M. P. M., Martinez, C. A., Costa, A. C.; Costa, P. M. F., Dietrich, S. M. C., & Buckeridge, M. S. Effect of atmospheric CO_2_ enrichment on the establishment of seedlings of Jatobá, *Hymenaea Courbaril* L. (Leguminosae, Caesalpinioideae). Biota Neotropica, 2,1–10, 2002. [https://doi.org/10.1590/S1676-06032002000100008](about:blank) (2002).

Approbato, A. U. Análises fisiológicas e bioquímicas da forrageira tropical Panicum maximum Jacq. (Poaceae) cultivada em elevado CO_2_ atmosférico e aquecimento. Tese de Doutorado, Faculdade de Filosofia, Ciências e Letras de Ribeirão Preto, Universidade de São Paulo, Ribeirão Preto. [https://doi.org/10.11606/T.59.2016.tde-04012016-141050](about:blank)., [www.teses.usp.br](about:blank) (2015).

Arenque, B. C. Metabolismo de carboidratos da espécie amazônica Senna reticulata sob cultivo em alto CO_2_. Dissertação de Mestrado, Instituto de Biociências, Universidade de São Paulo, São Paulo. doi:10.11606/D.41.2010.tde-30092010-151758. [www.teses.usp.br](about:blank) (2010).

Arenque-Musa, B. C., Grandis, A., Pocius, O., De Souza, A. P., Buckeridge, M. S. Responses of Senna reticulata, a legume tree from the Amazonian floodplains, to elevated atmospheric CO_2_ concentration and waterlogging. Trees, 28, 1021-1034. [https://doi.org/10.1007/s00468-014-1015-0](about:blank) (2014).

Arenque-Musa, B. C. Papel do metabolismo de carboidratos nas respostas ecofisiológicas da árvore amazônica Senna reticulata cultivada sob diferentes estresses abióticos. Tese de Doutorado, Instituto de Biociências, Universidade de São Paulo, São Paulo. doi:10.11606/T.41.2014.tde-29092014-101708. [www.teses.usp.br](about:blank) (2014).

Avila, R. T. Physiological and hydraulic mechanisms of drought tolerance of drought tolerance in plants: implications of CO_2_ and irradiance. Thesis (Plant Physiology Graduate Program) - Universidade Federal de Viçosa. [https://www.locus.ufv.br/bitstream/123456789/27778/3/texto%20completo.pdf](about:blank) (2020).

Baesso, R. C. E. Efeito do CO_2_ na eficiência quântica do eucalipto e sua utilização na modelagem de ser crescimento pelo 3-PG. Viçosa, MG. [https://www.locus.ufv.br/bitstream/123456789/1504/1/texto%20completo.pdf](about:blank) (2011).

Barbosa, M. L. Os efeitos positivos da aplicação do silício sobre a produção e desempenho fotossintético de plantas de arroz são anulados com o aumento da [CO_2_]. Thesis (Doutorado em Fisiologia Vegetal) - Universidade Federal de Viçosa, Viçosa. (2019).

Batista, E.R. Respostas fisiológicas e metabólicas de duas cultivares de *Coffea arabica* L. submetidas a atmosferas enriquecidas em CO_2_ em Câmaras de topo aberto e sistema FACE. Thesis. Instituto de Botânica da Secretaria de Estado do Meio Ambiente, 142 (2015).

Bianconi, M. E. Trocas gasosas foliares e crescimento de uma forrageira C4 tropical no contexto das mudanças climáticas: respostas ao elevado CO_2_ e ao aquecimento. (2014).

Bortolin, L.H.G.C. Simulação do clima de 2050 em campo e seus efeitos sobre o crescimento de forrageiras. 2016. Thesis(Doutorado em Ecologia e Recursos Naturais) – Universidade Federal de São Carlos, São Carlos. [https://repositorio.ufscar.br/handle/ufscar/8328](about:blank) (2016).

Braga, M., Aidar, M.PM., Marabesi, M.A., Godoy, JRL Effects of elevated CO_2_ on the phytoalexin production of two soybean cultivars differing in the resistance to stem canker disease, Environmental and Experimental Botany, 58,1–3, 85-92, [https://doi.org/10.1016/j.envexpbot.2005.06.018](about:blank) (2006).

Brito, M.N.O, Martinez, C.A.M, Mosquim, P.R., Silva, M.A., Oliva, M.A., Otoni, W. Crescimento e respostas fisiológicas de duas espécies de batata (Solanum sp.) sob ambientes enriquecidos com CO_2_. Dissertation (Mestrado em Ciências Agrárias (Fisiologia Vegetal)) - Universidade Federal de Viçosa. (1999).

Britto, A.P.C.H, Bortolin, L.H.G.C., Castro É., Martinez, C.A. (2016). Leaf Dynamics of *Panicum maximum* under Future Climatic Changes. PLoS ONE 11(2), [https://doi.org/10.1371/journal.pone.0149620](about:blank)

Costa, A.C Respostas fisiológicas de duas linhagens de soja à atmosfera enriquecida com CO_²_ e à restrição hídrica. Dissertation. (Mestrado em Fisiologia Vegetal) - Universidade Federal de Viçosa, Viçosa. [https://www.locus.ufv.br/handle/123456789/10023](about:blank) . (2003).

Costa, P. M. F. Efeitos da alta concentração de CO_2_ sobre o crescimento e o estabelecimento de plântulas do jatobá de mata *Hymeneae courbaril L*. Var. stilbocarpa (Heyne) Lee & Langenheim (Leguminosae, Caesalpinioideae, Detarieae). Dissertation (Mestrado em Biologia Celular e Estrutural) – Instituto de Biologia, Universidade Estadual de Campinas, Campinas (2004).

DaMatta, F.M., Godoy, A.G., Menezes-Silva, P.E., Martins, S.C.V., Sanglard, LMVP, Morais, L.E., Torre-Neto, A., Ghini, R. Sustained enhancement of photosynthesis in coffee trees grown under free-air CO_2_ enrichment conditions: disentangling the contributions of stomatal, mesophyll, and biochemical limitations. Journal of Experimental Botany, 67, 341-352, [https://doi.org/10.1093/jxb/erv463](about:blank) (2015).

Souza, A.P. A cana-de-açúcar e as mudanças climáticas: efeitos de uma atmosfera enriquecida com CO_2_ sobre o crescimento, desenvolvimento e metabolismo de carboidratos de *Saccharum ssp.* Tese apresentada ao Instituto de Biologia para obtenção do Título de Mestre em Biologia Celular e Estrutural na área de Biologia Celular. 88 (2007).

De Souza, A. P., Gaspar, M., Da Silva, E. A., Ulian, E. C., Waclawovsky, A. J., Nishiyama, JR., M. Y., Dos Santos, R. V., Teixeira, M. M., Souza, G. M., & Buckeridge, M. S. Elevated CO2 increases photosynthesis, biomass and productivity, and modifies gene expression in sugarcane. Plant, Cell & Environment, 31,1116-1127. [https://doi.org/10.1111/j.1365-3040.2008.01822.x](about:blank) (2008).

De Souza, A. P. Mecanismos fotossintéticos e relação fonte-dreno em cana-de-açúcar cultivadas em atmosfera eniquecida em CO_2_. Thesis. Instituto de Biociências da Universidade de São Paulo, Departamento de Botênica, 208, [https://www.teses.usp.br/teses/disponiveis/41/41132/tde-14092011-143401/publico/Amanda_Souza.pdf](about:blank) . (2011).

Souza, A. P. Mecanismos fotossintéticos e relação fonte-dreno em cana-de-açucar cultivada em atmosfera enriquecida de CO_2_. Doctoral Thesis, Instituto de Biociências, University of São Paulo, São Paulo. https:doi/10.11606/T.41.2011.tde-14092011-143401 (2011).

Dorneles, K., Celente, A.,Fernando, J., Amarante, L., Avila, L.,Deuner, S., Dallagnol, L. Increased atmospheric CO_2_ concentration causes modification of physiological, biochemical and histological characteristics that affects rice-Bipolaris oryzae interaction. European Journal of Plant Pathology. 157. 10.1007/s10658-020-01972-4 (2020).

Faria, A.P., Fernandes, G.W., França, M.G.C. Climate Change and Grass Seed Germination. Austral Ecology, 40: 962-973. [https://doi.org/10.1111/aec.12280](about:blank) (2015),

Fauset, S., Oliveira, L., Buckeridge, M.S., Foyer,C.H.,Galbraith, D., Tiwari, R., Gloor, M. Contrasting responses of stomatal conductance and photosynthetic capacity to warming and elevated CO_2_ in the tropical tree species Alchornea glandulosa under heatwave conditions, Environmental and Experimental Botany, 158, 28-39, [https://doi.org/10.1016/j.envexpbot.2018.10.030](about:blank). (2019).

Fontes, L. F. P. Respostas ecofisiológicas em árvores jovens de eucalipto ao enriquecimento atmosférico de CO_2_ e aquecimento em MINIFACE E MINI T-FACE.Thesis. (Doutorado em Engenharia Agrícola) - Universidade Federal de Viçosa, Viçosa.http://www.locus.ufv.br/handle/123456789/20534 (2017).

Ghini, R., Torre-Neto, A., Dentzien, A.F.M. et al. Coffee growth, pest and yield responses to free-air CO_2_ enrichment. Climatic Change 132, 307–320 [https://doi.org/10.1007/s10584-015-1422-2](about:blank) (2015).

Godoy, J.R.L. Ecofisiologia do estabelecimento de leguminosas arbóreas da Mata Atlântica, pertencentes a diferentes grupos funcionais, sob atmosfera enriquecida com CO_2_: uma abordagem sucessional. Thesis (Doutorado) -- Instituto de Botânica da Secretaria de Estado do Meio Ambiente (2007).

Grandis, A. Respostas fotossintéticas e de crescimento da espécie amazônica *Senna reticulata* sob elevada concentração de CO_2_. Dissertação de Mestrado, Instituto de Biociências, Universidade de São Paulo, São Paulo. doi:10.11606/D.41.2010.tde-18012011-171004 (2010).

Habermann, E., Oliveira, E. A. D., Contin, D. R., San Martin, J. A. B., Curtarelli, L., Gonzalez-Meler, M. A., & Martinez, C. A. Stomatal development and conductance of a tropical forage legume are regulated by elevated [CO_2_] under moderate warming. Frontiers in Plant Science, 10, 1–17. [https://doi.org/10.3389/fpls.2019.00609](about:blank) (2019).

Habermann, E., San Martin, J.A.B., Contin, D.R., Bossan, V.P., Barboza, A., Braga, M.R., et al. Correction: Increasing atmospheric CO_2_ and canopy temperature induces anatomical and physiological changes in leaves of the C4 forage species *Panicum maximum*. PLoS ONE 15(8), [https://doi.org/10.1371/journal.pone.0238275](about:blank) (2020)

Kretzschmar, F.S., Aidar, M.P.M., Salgado, I., Braga, M.R. Elevated CO_2_ atmosphere enhances production of defense-related flavonoids in soybean elicited by NO and a fungal elicitor, Environmental and Experimental Botany, 65(2–3), 319-329, [https://doi.org/10.1016/j.envexpbot.2008.10.001](about:blank) (2019).

Kretzchmar, F.S. Influencia de atmosfera enriquecida em CO_2_, do radical oxido nitrico e de eliciadores fungicos na produção de fitoalexinas em plantulas de soja. Dissertation (mestrado) - Universidade Estadual de Campinas, Instituto de Biologia, Campinas, SP. 118. https://hdl.handle.net/20.500.12733/1605203 (2007).

Lobo, F.A. Alterações na assimilação e metabolismo do carbono em plantas de soja sob condições de atmosfera enriquecida com CO_2_.Thesis (Doutorado em Fisiologia Vegetal) - Universidade Federal de Viçosa, Viçosa, 105 (2003).

Machado, M. R. Estudo bioquimico e da estrutura foliar de plântulas do jatobá da mata (Hymenaea courbaril L.) e do cerrado (*Hymenaea stigonocarpa* M.) expostas à concentração elevada de CO_2_. Tese -Universidade Estadual de Campinas, Instituto de Biologia (2007).

Marabesi, M. A. Efeito do alto CO_2_ no crescimento inicial e na fisiologia da fotossíntese em plântulas *Senna alata* (L ) Roxb Dissertação. Instituto de Botânica da Secretaria do Meio Ambiente. 1–78 (2007).

Marçal, R.T., Dinorah, M.S., Avila, L. F. Quiroga-Rojas, Souza, R.P.B., Gomes C.C.J., Ponte, L.R., Barbosa, M.L.,Oliveira, L.A., Martins, S.C.V., Ramalho, J.D.C., DaMatta, F.M. Elevated [CO_2_] benefits coffee growth and photosynthetic performance regardless of light availability,Plant Physiology and Biochemistry, 158, 524-535, [https://doi.org/10.1016/j.plaphy.2020.11.042](about:blank) (2021).

Martins, M. Q., Rodrigues, W. P., Fortunato, A. S., Leitão, A. E., Rodrigues, A. P., Pais, I. P., Martins, L. D., Silva, M. J., Reboredo, F. H., Partelli, F. L., Campostrini, E., Tomaz, M. A., Scotti-Campos, P., Ribeiro-Barros, A. I., Lidon, F. C., DaMatta, F. M., Ramalho J. C. Protective response mechanisms to heat stress in interaction with high [CO_2_] conditions in Coffea spp. Frontiers in Plant Science, 7:947 (2016)

Mayorga, A.Y. Desenvolvimento e efeito da concentração atmosférica de CO_2_ e da temperatura em plântulas juvenis de Hymenaea courbaril L., jatobá. Tese de Doutorado, Instituto de Biociências, Universidade de São Paulo, São Paulo. https://10.11606/T.41.2010.tde-21022011-134614 (2010).

Melo, N.M.J. Respostas ecofisiológicas de plantas ocorrentes no cerrado frente à elevada concentração de CO_2_. Dissertation (Mestrado em Manejo e Conservação de Ecossistemas Naturais e Agrários) - Universidade Federal de Viçosa, Florestal. 76. (2015).

Melo, N.M.J., Rosa Rayete S.-E. G., Pereira, E.G., Souza, J.P. Rising [CO_2_] changes competition relationships between native woody and alien herbaceous Cerrado species. Functional Plant Biology 45, 854-864. [https://doi.org/10.1071/FP17333](about:blank) (2018)

Melo, N.M.J. Morfologia da copa e crescimento de espécies arbóreas de cerrado em campo e em câmaras de topo aberto sob elevada cncentração de CO_2_. Thesis ( Programa de Pós-Graduação em Ecologia e Recursos Naturais do Centro de Ciências Biológicas e da Saúde da Universidade Federal de São Carlos), [https://repositorio.ufscar.br/bitstream/handle/ufscar/12978/Tese_NayaraMagryJesusMelo_26-06-2020-.pdf?sequence=3&isAllowed=y](about:blank) (2020).

Mendes de Sá, C.E., Negreiros, D., Fernandes, G.W., Dias, M.C., Franco, A.C. Carbon dioxide-enriched atmosphere enhances biomass accumulation and meristem production in the pioneer shrub *Baccharis dracunculifolia (*Asteraceae). Acta Botanica Brasilica [online]. 28 (4), pp. 646-650. https://doi.org/10.1590/0102-33062014abb3329 (2014).

Miroslava, R., Ribeiro, R.V., Marchiori, P. E. R., Filizola, H.F., Batista, E.R. Structural and functional changes in coffee trees after 4 years under free air CO_2_ enrichment, Annals of Botany,121,5, 1065–1078, [https://doi.org/10.1093/aob/mcy011](about:blank) (2018).

Mortari, L. C. Efeitos de uma atmosfera enriquecida com CO_2_ sobre a fotossíntese, o crescimento e o metabolismo de carboidratos do açaí (*Euterpe oleracea* Mart.). Dissertation. Universidade de São Paulo, São Paulo. [https://doi:10.11606/D.41.2012.tde-01052013-113711](about:blank), (2012).

Oliveira, V. F. Efeito da atmosfera enriquecida em CO_2_ no crescimento, na alocação de biomassa e no metabolismo de frutanos em Vernonia herbacea (Vell.) Rusby - São Paulo, 79 (2007).

Oliveira, V.F., Zaidan, L. B. P., Braga M. R., Aidar, M.P. M., Carvalho M.A M. Elevated CO_2_ atmosphere promotes plant growth and inulin production in the cerrado species *Vernonia herbacea*. Functional Plant Biology 37, 223-231. [https://doi.org/10.1071/FP09164](about:blank) (2010)

Oliveira, E.A.D., Approbato, A.U., Legracie, J.R., Martinez, C.A. Soil-nutrient availability modifies the response of young pioneer and late successional trees to elevated carbon dioxide in a Brazilian tropical environment, Environmental and Experimental Botany, 77, 53-62, [https://doi.org/10.1016/j.envexpbot.2011.11.003](about:blank) (2012).

Oliveira, V.F., Silva, E.A., Zaidan, L.B.P., Carvalho, M.A.M. Effects of elevated CO_2_ concentration and water deficit on fructan metabolism in Viguiera discolor Baker. Plant Biology, 15, 471-482. [https://doi.org/10.1111/j.1438-8677.2012.00654.x](about:blank) (2013),

Oliveira, V. F., Silva, E.A., Carvalho, M.A.M. Elevated CO_2_ Atmosphere Minimizes the Effect of Drought on the Cerrado Species *Chrysolaena obovata*. Frontiers in Plant Science. https://doi/10.3389/fpls.2016.00810 (2016).

Oliveira, E.A.D. Impacto de elevadas concentrações de CO_2_ e da fertilidade do solo sobre a fisiologia e crescimento inicial de *Croton urucurana* Baill e *Cariniana legalis* (Mart.) Kuntze, numa simulação climática futura . (2008).

Olivo, N., Martinez, C., Oliva, M. The Photosynthetic Response to Elevated CO_2_ in High Altitude Potato Species (*Solanum curtilobum*). *Photosynthetica* 40, 309–313. [https://doi.org/10.1023/A:1021370429699](about:blank) (2002).

Pimenta, T.M. Aspectos anatômicos e fisiológicos de plantas de tomate associados a inibição da biossíntese de giberelinas e à elevada concentração de dióxido de carbono. Dissertation. (Mestrado em Fisiologia Vegetal) - Universidade Federal de Viçosa, Viçosa. 37 (2017).

Rezende, L. F.C. Análise e modelagem de ecofisiologia da espécie *Poincianella microphylla* da Caatinga em campo e sob condições de alta concentração de CO_2_.Thesis (Doutorado em Ciência do Sistema Terrestre) – Instituto Nacional de Pesquisas Espaciais, São José dos Campos, 147 (2015).

Pereira, R. W. Effect of high temperatures and CO_2_ concentration on physiological, biochemical and growth traits in *Coffea sp.* : aspects related to the single leaf and whole canopy. Thesis (Doutorado em Produção Vegetal) -- Universidade Estadual do Norte Fluminense Darcy Ribeiro. Centro de Ciências e Tecnologias Agropecuárias. Laboratório de Melhoramento Genético Vegetal. Campos dos Goytacazes, 107 (2017).

Silva, J.B.LCrops answer of beans subjected to water stress and corn in an atmosphere enriched with CO_2_. Thesis. (Doutorado em Construções rurais e ambiência; Energia na agricultura; Mecanização agrícola; Processamento de produção) - Universidade Federal de Viçosa, Viçosa, Retrieved 2022-10-28, from [http://bdtd.ibict.br/vufind/Record/UFV_72ca743d2a831230b77659ad5c7e59e9](about:blank) .(2010).
